# Functionalized Lanthanide Oxide Nanoparticles for Tumor Targeting, Medical Imaging, and Therapy

**DOI:** 10.3390/pharmaceutics13111890

**Published:** 2021-11-08

**Authors:** Mohammad Yaseen Ahmad, Huan Yue, Tirusew Tegafaw, Shuwen Liu, Son Long Ho, Gang Ho Lee, Sung-Wook Nam, Yongmin Chang

**Affiliations:** 1 Department of Chemistry, College of Natural Sciences, Kyungpook National University, Taegu 41566, Korea; yaseen.knu@gmail.com (M.Y.A.); yuehuan888@gmail.com (H.Y.); tirukorea@gmail.com (T.T.); liushuwen0701@gmail.com (S.L.); sonlongh@gmail.com (S.L.H.); 2 Department of Molecular Medicine, School of Medicine, Kyungpook National University, Taegu 41405, Korea; nams@knu.ac.kr

**Keywords:** imaging agent, lanthanide oxide nanoparticle, tumor targeting, toxicity

## Abstract

Recent progress in functionalized lanthanide oxide (Ln_2_O_3_) nanoparticles for tumor targeting, medical imaging, and therapy is reviewed. Among the medical imaging techniques, magnetic resonance imaging (MRI) is an important noninvasive imaging tool for tumor diagnosis due to its high spatial resolution and excellent imaging contrast, especially when contrast agents are used. However, commercially available low-molecular-weight MRI contrast agents exhibit several shortcomings, such as nonspecificity for the tissue of interest and rapid excretion in vivo. Recently, nanoparticle-based MRI contrast agents have become a hot research topic in biomedical imaging due to their high performance, easy surface functionalization, and low toxicity. Among them, functionalized Ln_2_O_3_ nanoparticles are applicable as MRI contrast agents for tumor-targeting and nontumor-targeting imaging and image-guided tumor therapy. Primarily, Gd_2_O_3_ nanoparticles have been intensively investigated as tumor-targeting T_1_ MRI contrast agents. T_2_ MRI is also possible due to the appreciable paramagnetic moments of Ln_2_O_3_ nanoparticles (Ln = Dy, Ho, and Tb) at room temperature arising from the nonzero orbital motion of 4f electrons. In addition, Ln_2_O_3_ nanoparticles are eligible as X-ray computed tomography contrast agents because of their high X-ray attenuation power. Since nanoparticle toxicity is of great concern, recent toxicity studies on Ln_2_O_3_ nanoparticles are also discussed.

## 1. Introduction

Medical imaging plays an important role in the pre-detection, diagnosis, and treatment of tumors [1]. Among the currently available medical imaging techniques, magnetic resonance imaging (MRI) allows whole-body imaging and outstanding microsoft tissue contrast and image resolution to reveal morphological and anatomical details of tissues [2,3]. As imaging agents, various nanoparticles have been developed due to their remarkable physical and chemical properties, which are superior to those of small molecules [4,5,6,7]. Moreover, nanoparticles can be easily surface-functionalized for advanced imaging and tumor targeting [8,9]. They can also provide longer blood circulation times than small molecules, which is conducive to tumor targeting and drug delivery to specific tumor cells [10,11].

Lanthanide oxide (Ln_2_O_3_) nanoparticles (Ln = Gd, Tb, Dy, and Ho) are of special interest because they have appreciable magnetic moments at room temperature, which is useful for MRI [12,13,14,15], and high X-ray attenuation power, which is useful for X-ray computed tomography (CT) [16,17,18]. In addition, surface-modified Ln_2_O_3_ nanoparticles exhibit improved properties, such as high-water proton spin relaxivities, high colloidal stabilities, and low toxicities [12,13,14]. For in vivo applications, nanoparticles should have an ultrasmall particle diameter (<3 nm) to allow their excretion from the body via the urinary system after intravenous injection [19,20]. Ln_2_O_3_ nanoparticles meet such requirements, displaying excellent MRI and CT imaging properties at ultrasmall particle diameters [12,13,14,15].

The development of tumor-targeting Ln_2_O_3_ nanoparticles is challenging. Especially when compared with commercial molecular Gd-chelates [21,22,23], Gd_2_O_3_ nanoparticles are more efficient longitudinal relaxation promoters [12,13,14,15] because their longitudinal relaxivity (r_1_) values are higher than those (i.e., 3–5 s^‒1^mM^‒1^) [21,22,23] of commercial molecular MRI contrast agents. Therefore, Gd_2_O_3_ nanoparticles can provide very high contrast T_1_ MR images and thus, are ideal candidates for tumor-targeting T_1_ MRI contrast agents. In particular, their r_1_ value is optimal at ultrasmall nanoparticle size (1.0–2.5 nm) [24,25]. Meanwhile, other Ln_2_O_3_ nanoparticles (Ln = Dy, Tb, and Ho) are eligible as T_2_ MRI contrast agents [12,13,15].

Nanoparticles alone accumulate nonspecifically in tumors via passive targeting, i.e., the enhanced permeability and retention (EPR) effect [26]. The accumulation amount and specificity to tumors can be enhanced by active targeting, which is commonly achieved by modifying contrast agents with tumor-targeting ligands that can selectively bind to receptors overexpressed on tumor-cell membranes. Such tumor-targeting ligands include small molecules, such as arginylglycylaspartic acids (Arg-Gly-Asp or RGDs) [27,28,29] and folic acid [30,31], peptides, such as chlorotoxin (CTX) [32,33], and biological molecules, such as antibodies [34]. Nanoparticles can provide a flexible platform to attach tumor-targeting ligands, thereby improving their specificity and effectiveness for tumor treatment. In addition, anticancer drugs can also be attached to nanoparticle surfaces for chemotherapy. Generally, in this type of treatment, most anticancer drugs cannot differentiate between tumor and normal cells, causing toxic side effects [35,36]. However, such side effects can be minimized or eliminated by delivering drugs via tumor-targeting nanoparticles. However, the cytotoxicity and biocompatibility of lanthanides during and after endocytosis by cells are still largely unknown, although numerous reports describe lanthanides as relatively non-toxic elements [37,38].

The main purpose of this review is to provide an insight into tumor imaging and image-guided tumor therapy using functionalized (i.e., tumor-targeting ligand-conjugated) Ln_2_O_3_ nanoparticles. First, nanoparticle synthesis methods, surface-modification, tumor-targeting ligand conjugation, and various experimental analyses for Ln_2_O_3_ nanoparticles are introduced. Second, physicochemical properties, such as particle diameters and magnetic properties, and imaging properties, including relaxivities, are discussed. Third, recent developments of tumor-targeting T_1_ MRI contrast agents based on Gd_2_O_3_ nanoparticles that were applied in tumor imaging and image-guided tumor therapy and Ln_2_O_3_ nanoparticle-based T_2_ MRI and CT contrast agents are discussed. Finally, recent progress in the knowledge of the toxicity of Ln_2_O_3_ nanoparticles is discussed. Overall, this review provides an overview of the recent progress in functionalized Ln_2_O_3_ nanoparticles applied in tumor imaging and therapy.

## 2. Synthesis and Surface Functionalization of Ln_2_O_3_ Nanoparticles

**Figure 1:**
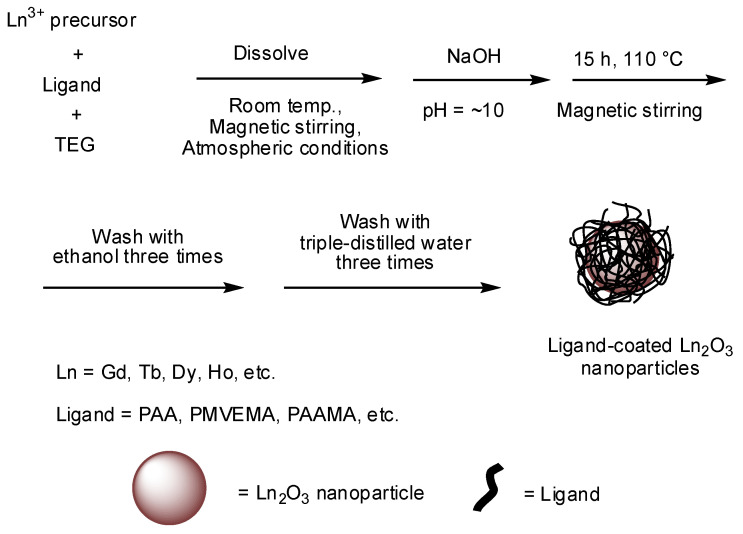
Reaction scheme for the one-pot polyol synthesis of hydrophilic and biocompatible polymer-coated ultrasmall Ln_2_O_3_ nanoparticles. TEG = triethylene glycol.

Among the various methods currently available for the synthesis of ultrasmall Ln_2_O_3_ nanoparticles, the synthesis in a polyol solvent is preferred for biomedical applications because ultrasmall nanoparticles are obtained (average particle diameter = 2.0 nm) and subsequent surface coating of the nanoparticles with hydrophilic and biocompatible ligands can be performed in one pot [17,31]. A general reaction scheme for the polyol synthesis is provided in Figure 1.

**Figure 2:**
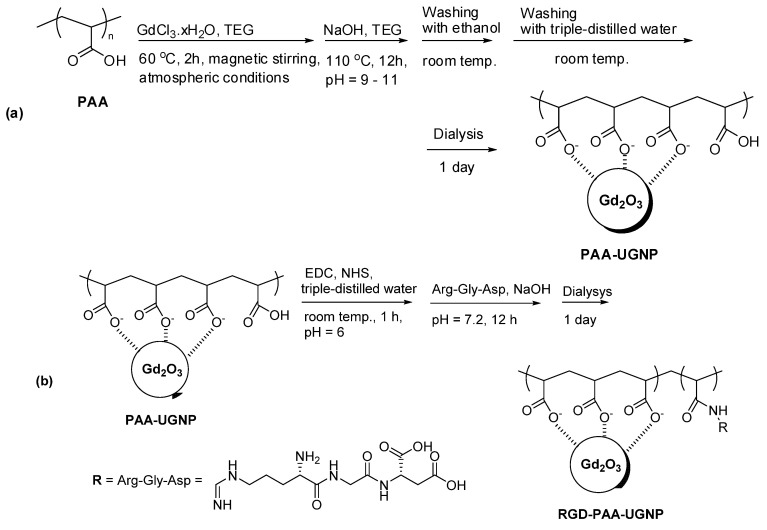
Two-step synthesis of RGD-PAA-Gd_2_O_3_ nanoparticles. (**a**) Step 1: PAA-Gd_2_O_3_ nanoparticles and (**b**) step 2: conjugation of RGD with PAA-Gd_2_O_3_ nanoparticles. EDC = *N*-(3-dimethylaminopropyl)-*N*′-ethylcarbodiimide, NHS = *N*-hydroxysuccinimide, and UGNP = ultrasmall gadolinium oxide nanoparticle. Adapted with permission from [44], The Royal Society of Chemistry, 2020.

Nanoparticle contrast agents possess an additional advantage over molecular agents because cancer-targeting ligands and drugs can be easily attached to the nanoparticle surfaces. Small molecular ligands are less efficient in providing good colloidal stability to nanoparticles compared with polymer ligands, which is due to the presence of many hydrophilic binding groups in the polymers for attachment to the nanoparticles [39,40,41,42,43]. In addition, hydrophilic polymers can provide higher r_1_ values than small molecular ligands [39,40,41] because they can attract more water molecules around the nanoparticles. Examples of these polymers are polyacrylic acid (PAA), polymethyl vinyl ether-alt-maleic acid (PMVEMA), and polyacrylic acid-co-maleic acid (PAAMA) having numerous COOH groups (Figure 1) [39,40,41], which can serve as anchor groups of functional molecules, such as cancer-targeting ligands and drugs. For example, RGD-conjugated PAA-coated Gd_2_O_3_ nanoparticles were reported by Ho et al. [44] (Figure 2).

**Figure 3:**
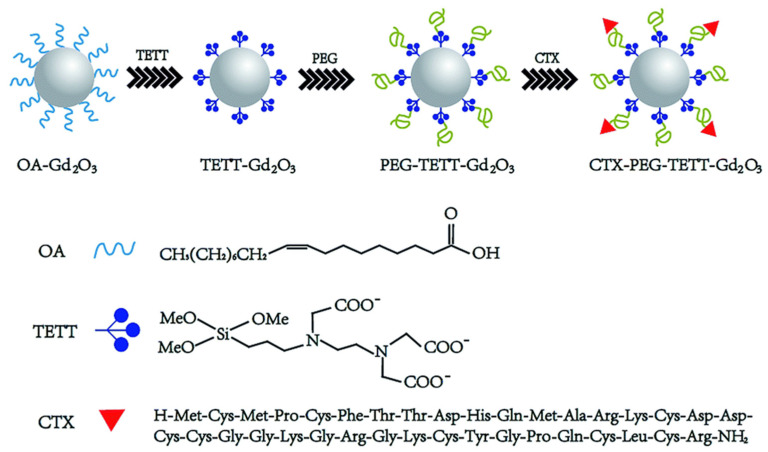
Schematic illustration for the synthesis of CTX-PEG-TETT-Gd_2_O_3_ nanoparticles. TETT = *N*-(trimethoxysilylpropyl) ethylenediamine triacetic acid trisodium salt and OA = oleic acid. Reproduced from [45], The Royal Society of Chemistry, 2014.

Ln_2_O_3_ nanoparticles can also be synthesized in organic solvents via the thermal decomposition method. The synthesized nanoparticles can be further coated with hydrophilic ligands and then conjugated with cancer-targeting ligands. For example, CTX-poly(ethylene glycol)-*N*-(trimethoxysilylpropyl) ethylenediamine triacetic acid silane-coated Gd_2_O_3_ nanoparticles (CTX-PEG-TETT-Gd_2_O_3_) were reported by Gu et al. [45] as tumor-targeting contrast agents (Figure 3). Here, the biocompatible poly(ethylene glycol) (PEG) layer is known to stabilize the Gd_2_O_3_ nanoparticles, enhance blood circulation blood times, and improve colloidal stability [11].

## 3. Physicochemical Characterization

Various experimental techniques can be used to characterize the synthesized Ln_2_O_3_ nanoparticles [17,31], as summarized in Table 1. The particle diameters, the hydrodynamic diameters of nanoparticles dispersed in water, and the crystal structure of the nanoparticles can be measured using a high-resolution transmission electron microscope (HRTEM) operating at high acceleration voltages (>200 kV), a dynamic light scattering (DLS) particle size analyzer, and a powder X-ray diffraction (XRD) spectrometer, respectively. The attachment of ligands on the nanoparticle surfaces can be investigated by recording Fourier transform-infrared (FT-IR) absorption spectra using powder samples; powder samples may be pelletized in KBr for easy handling and acquisition of good signal-to-noise ratios using a small number of powder samples. The amount of surface-coating ligand can be estimated according to thermogravimetric analysis (TGA) curves. The elemental analysis (EA) can also be used to investigate the surface-coating amount and ligand composition (C/H/O/N/S) in metal oxide nanoparticles because metal oxide nanoparticles hardly decompose during EA. The Ln-concentration in an aqueous nanoparticle suspension can be determined using an inductively coupled plasma atomic emission spectrometer (ICPAES). The in vitro cellular cytotoxicity of the ligand-coated Ln_2_O_3_ nanoparticles can be measured using various cell viability assay techniques, such as trypan blue dye exclusion staining, methylthiazoly tetrazolium (MTT) reduction, and water-soluble tetrazolium salt (WST) assay [46]. Cell viability measurements are generally performed using more than two cell lines to obtain consistent and reliable results because the cell viabilities generally depend on the cell lines. Magnetization (M) values of the nanoparticles can be estimated by recording M versus applied field (H) (M−H) curves at room temperature using a vibrating sample magnetometer (VSM). Net M values of the nanoparticles without ligand can be obtained using the net mass of the nanoparticles determined via TGA. For MRI contrast agents, r_1_ and the transverse relaxivity (r_2_) represent their ability to act as T_1_ or T_2_ MRI contrast agents. To measure the r_1_ and r_2_ values, longitudinal (T_1_) and transverse (T_2_) relaxation times are measured using an MRI scanner. The T_1_ relaxation time measurements can be conducted using an inversion recovery method. For the T_2_ relaxation times, the Carr–Purcell Meiboom–Gill pulse sequence can be used for multiple spin-echo measurements. The r_1_ and r_2_ values are then estimated from the slopes of the plots of 1/T_1_ and 1/T_2_ versus the Ln-concentration, respectively. In vivo T_1_ or T_2_ MR images can be obtained using an MRI scanner after intravenous injection of an aqueous nanoparticle suspension sample. In addition, the X-ray attenuation power of Ln_2_O_3_ nanoparticles, which is higher than those of commercial iodine CT contrast agents, can be measured by recording phantom images using a CT scanner, and in vivo CT images can be obtained using a CT scanner after intravenous injection of an aqueous nanoparticle suspension sample.

## 4. Physicochemical and Imaging Properties

### 4.1. Particle Diameters and Surface Coatings

**Figure 4:**
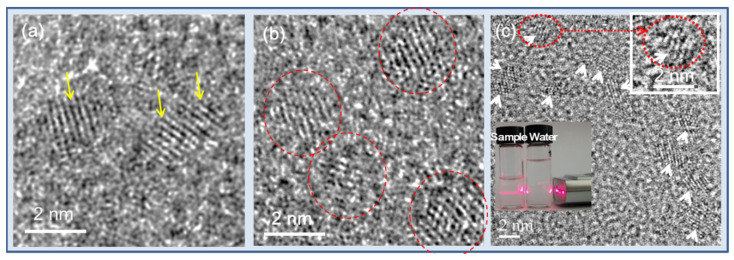
(**a**–**c**) HRTEM images [44,47,48]. Arrows and circles indicate nanoparticles. Insets in (**c**) show a magnified image of a nanoparticle (top right) and laser light scattering (i.e., Tyndall effect) due to the collision of the nanoparticle colloids with a passing laser light, whereas triple-distilled water shows no light scattering, confirming the good colloidal dispersion of nanoparticles in aqueous media (bottom left). Reproduced from [44,47,48]. Copyright 2020 The Royal Society of Chemistry [44]; Copyrights 2018 Wiley [47,48].

The imaging properties of nanoparticles depend on their particle and hydrodynamic diameters. Ln_2_O_3_ nanoparticles synthesized via the polyol method generally have ultrasmall diameters (average particle diameter = 2.0 nm) [39,40,41,42,43,44]. Figure 4a–c shows various HRTEM images of Gd_2_O_3_ nanoparticles [44,47,48]. Both particle and hydrodynamic diameters follow a log-normal function distribution; therefore, average particle and hydrodynamic diameters can be obtained by fitting the log-normal distribution function to the observed particle and hydrodynamic diameter distributions, respectively. The insets in Figure 4c show a nanoparticle suspension sample exhibiting laser light scattering (i.e., Tyndall effect) due to the collision of the nanoparticle colloids with the passing laser light, whereas triple-distilled water shows no light scattering [44]. This experiment shows the good colloidal dispersion of the nanoparticles in aqueous media.

**Figure 5:**
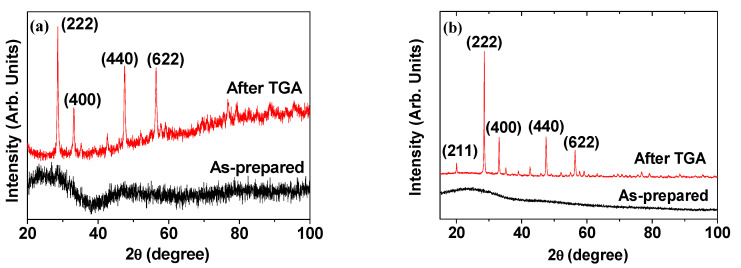
XRD patterns of (**a**) cRGD-Gd_2_O_3_ nanoparticles [47] and (**b**) RGD-PAA-Gd_2_O_3_ nanoparticles [44] before (i.e., as-prepared; bottom figures) and after TGA (top figures). Adapted from [44,47]. Copyright 2020 The Royal Society of Chemistry [44]; Copyright 2018 Wiley [47].

Due to the ultrasmall particle diameters, the XRD patterns of nanoparticle powder samples are generally broad and amorphous [49,50]. Nevertheless, upon performing a TGA from room temperature to high temperatures (>500 °C), sharp peaks corresponding to the (222), (400), (440), and (622) planes of a highly crystallized form of Ln_2_O_3_ having cubic structure are observed [50]. Examples of XRD patterns of ligand-coated Gd_2_O_3_ nanoparticles recorded before (i.e., as-prepared) and after TGA [44,47] are provided in Figure 5. The peak positions and the estimated cell constants of the TGA-analyzed samples are well consistent with those given in the literature [51]. The sharp peaks observed in the XRD patterns after TGA are due to the particle size growth and subsequent crystallization of the nanoparticles (Figure 5a,b), which was confirmed by Kattel et al. on the basis of HRTEM images [50,52].

**Figure 6:**
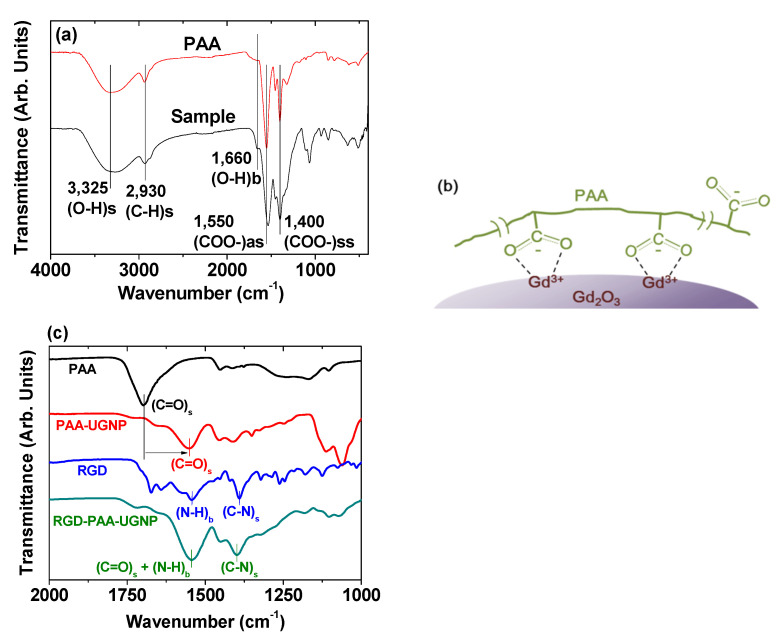
(**a**) FT-IR absorption spectra of PAA-coated Gd_2_O_3_ nanoparticles and free PAA sodium salt (M_w_ = 5100 Da) and (**b**) surface-coating structure of PAA on the Gd_2_O_3_ nanoparticle surface [39]. (**c**) FT-IR absorption spectra of PAA (M_w_ = 1800 Da), PAA-Gd_2_O_3_ nanoparticles, RGD, and RGD-PAA-Gd_2_O_3_ nanoparticles [44]. The lowercase letters “s”, “ss”, “as”, and “b” indicate stretch, symmetric stretch, antisymmetric stretch, and bend, respectively. Adapted from [39,44]. Copyrights 2018 and 2020 The Royal Society of Chemistry, respectively.

Surface coating of Ln_2_O_3_ nanoparticles with hydrophilic and biocompatible ligands is essential for their biomedical applications. The surface coating can be investigated by recording FT-IR absorption spectra. An FT-IR absorption spectrum of PAA-coated Gd_2_O_3_ nanoparticles is provided as an example at the bottom of Figure 6a [39], along with an FT-IR absorption spectrum of PAA (M_w_ = 5100 Da) at the top of the same figure as a reference. The spectrum of the PAA-coated Gd_2_O_3_ nanoparticles exhibited a series of bands stemming from PAA, such as C−H stretching (2930 cm^−1^), COO^−^ antisymmetric stretching (1550 cm^−1^), and COO^−^ symmetric stretching vibrations (1400 cm^−1^). The PAA and the nanoparticle were bonded through a coordination bonding between Gd^3+^ on the nanoparticle surface as a hard acid and the COO^−^ group of PAA as a hard base [53,54,55]. In this case, a stable nanoparticle colloid was formed in aqueous media because of the multiple bonding between many COO^−^ groups of PAA (each PAA has ~54 COO^−^ groups) and a nanoparticle. On this basis, the bonding between PAA and the Gd_2_O_3_ nanoparticle surface was proposed, as displayed in Figure 6b. Another example of surface coating on Gd_2_O_3_ nanoparticles is provided in Figure 6c, which displayed the FT-IR absorption spectra of RGD, PAA (M_w_ = 1800 Da), PAA-Gd_2_O_3_, and RGD-PAA-Gd_2_O_3_ powder samples [44]. The presence of a C=O stretching band (1553 cm^−1^) of PAA in the FT-IR absorption spectrum of PAA-Gd_2_O_3_ nanoparticles confirmed the PAA surface coating on the Gd_2_O_3_ nanoparticles. This band was red-shifted by 144 cm^−1^ compared with free PAA (1697 cm^−1^), which can be attributed to the coordination bonding between the COO^−^ groups of PAA and the Gd^3+^ of the Gd_2_O_3_ nanoparticles. As mentioned above, each PAA can form many coordination bonds with each Gd_2_O_3_ nanoparticle via a hard acid (Gd^3+^)–hard base (COO^−^) interaction [53,54,55]. The N−H bending vibration at 1544 cm^−1^ and the C–N stretching vibration at 1390 cm^−1^ of RGD [56] appeared in the FT-IR absorption spectrum of the RGD-PAA-Gd_2_O_3_ nanoparticles, confirming the successful conjugation of RGD to PAA in the PAA-Gd_2_O_3_ nanoparticles via amide bond formation.

**Figure 7:**
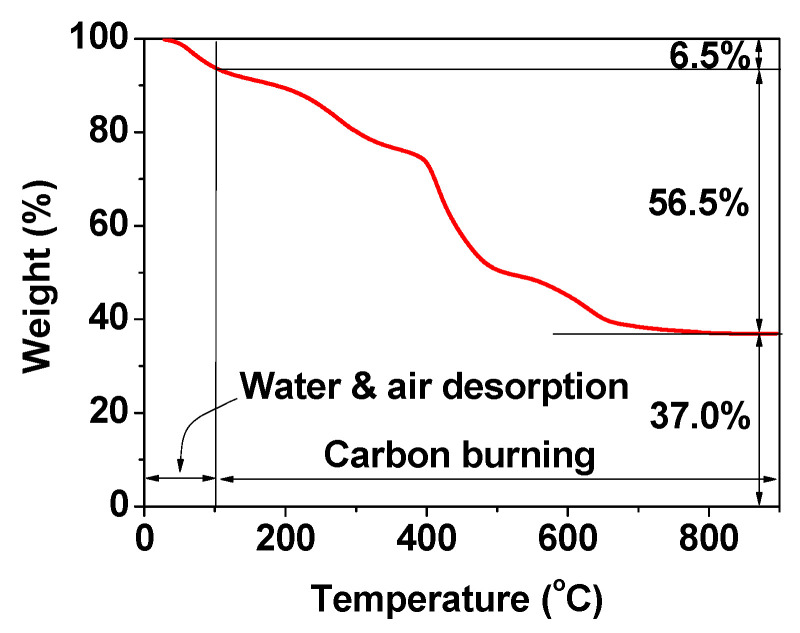
TGA curve of the powder sample of C@Gd_2_O_3_ nanoparticles. Adapted from [57]. Copyright 2019 Elsevier.

The EA can also be used to investigate the surface coating of nanoparticles. For example, in carbon-coated Gd_2_O_3_ nanoparticles (C@Gd_2_O_3_), the C/H/O content, in which H mostly stems from hydrocarbons and -OH groups and O mostly stems from -OH groups on the carbon-coating layer, was determined to be 28.1/3.2/26.5 in wt% [57]. The sum of these wt% is 57.8%, which is fairly consistent with the 56.5% estimated from the TGA curve (Figure 7). As can be seen in Figure 7, the remaining 37.0% corresponds to the mass wt% of Gd_2_O_3_ in the C@Gd_2_O_3_ nanoparticles. By performing a grafting density analysis [58], the wt% can be converted into the number of ligands grafted onto each nanoparticle surface [47,48].

### 4.2. Magnetic Properties

**Figure 8:**
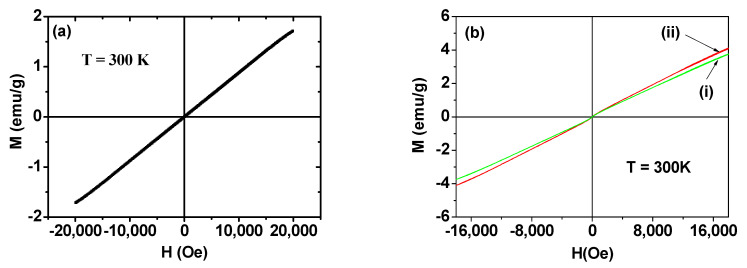
(**a**) M–H curve of Gd_2_O_3_ nanoparticles at T = 300 K [41]. (**b**) M–H curves of Ln_2_O_3_ nanoparticles, Ln = (i) Tb and (ii) Ho at T = 300 K [43]. Net M values of Ln_2_O_3_ nanoparticles without ligands were used in the plots. Adapted from [41,43]. Copyrights 2020 and 2021 MDPI.

To obtain high r_1_ and r_2_ values for enhanced MR contrast images, the nanoparticles must exhibit large M values at room temperature [59,60]. Figure 8a,b shows the M–H curves of Ln_2_O_3_ nanoparticles (Ln = Gd, Tb, Ho) [41,43]. Similar to the corresponding bulk materials [61,62,63], all of the nanoparticle samples are paramagnetic (i.e., no hysteresis, zero coercivity, zero remanence, and low M values) and exhibit appreciable unsaturated M values up to the measured H values at 300 K. From the mass-corrected M–H curve, the net M value of the Gd_2_O_3_ nanoparticles, which corresponds to the nanoparticles without ligands, at 2.0 T was estimated to be 1.71 emu/g. This appreciable value is due to the high 4f-electron spin magnetic moment (s = 7/2) of Gd^3+^ having seven unpaired 4f electrons. Meanwhile, other Ln_2_O_3_ nanoparticles (Ln = Tb and Ho) exhibit net M values of 3.8 and 4.1 emu/g at 1.8 T (Figure 8b), respectively, which result from high 4f-electron spin–orbital magnetic moments, i.e., J = 6 for Tb^3+^ and J = 8 for Ho^3+^. The slightly higher M value of the Ho_2_O_3_ nanoparticles compared with that of the Tb_2_O_3_ nanoparticles is due to the higher atomic magnetic moment (µ = 10.60 µ_B_) of Ho^3+^ compared with that of Tb^3+^ (= 9.72 µ_B_) [64], where µ_B_ is the Bohr magneton.

### 4.3. MR Imaging Properties: r_1_ and r_2_ Values

**Figure 9:**
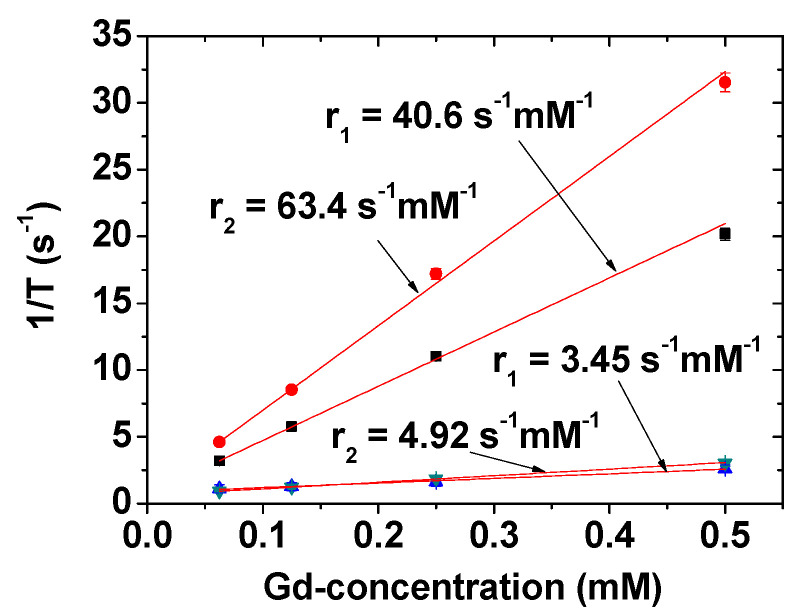
r_1_ and r_2_ values of PAAMA-coated Gd_2_O_3_ nanoparticles [40]. Adapted from [40]. Copyright 2021 MDPI.

The r_1_ and r_2_ values are affected by the number of water molecules around the nanoparticles and the distance between them and, therefore, depend on the water-attracting ability of the ligands around the nanoparticles [59,60,65]. Accordingly, surface-coating ligands should be properly selected. Gd_2_O_3_ nanoparticles act as positive (T_1_) MRI contrast agents due to the high spin magnetic moment of Gd^3+^ (s = 7/2); they have high r_1_ values and r_2_/r_1_ ratios close to one [39,40,41] (T_1_ MRI contrast agents exhibit low r_2_/r_1_ ratios, whereas T_2_ MRI contrast agents have high r_2_/r_1_ ratios [23]). The r_1_ and r_2_ values of PAAMA-coated Gd_2_O_3_ nanoparticles at 3.0 T MR field were determined to be 40.6 and 63.4 s^−1^ mM^−1^ (r_2_/r_1_ = 1.56), respectively, using the 1/T_1_ and 1/T_2_ plots versus Gd-concentration depicted in Figure 9 [40]. This r_1_ value is approximately 10 times higher than those [21,22,23] of commercial molecular T_1_ MRI contrast agents, such as Dotarem, ProHance, Gadovist, Magnevist, and Omniscan [21,22]. The r_1_ value also depends on the particle diameter of Gd_2_O_3_ nanoparticles, for which the optimal value was suggested to be 1.0 to 2.5 nm [24]. The observed particle diameter of the PAAMA-coated Gd_2_O_3_ nanoparticles is within this size range. As a reference, the r_1_ and r_2_ values of Gadovist as a commercial molecular agent are provided at the bottom of Figure 9, which shows that this nanoparticle sample is more powerful than Gadovist as a T_1_ MRI contrast agent. On the other hand, other Ln_2_O_3_ nanoparticles (Ln = Tb, Dy, and Ho) show appreciable to high r_2_ values and negligible r_1_ values with very large r_2_/r_1_ ratios [66]. Therefore, these nanoparticles are suitable as T_2_ MRI contrast agents. The r_2_ values of these nanoparticles increased with increasing the MR field [66] because r_2_ is proportional M^2^ [59,60]. Therefore, these nanoparticles are expected to provide appreciable T_2_ MR contrast images at high MR fields. For example, PAA-coated Ln_2_O_3_ nanoparticles (Ln = Tb and Ho) exhibit appreciable T_2_ MR contrast images at 9.4 T MR Field [43]. For comparison, this T_2_ contrast is lower than that of superparamagnetic iron oxide nanoparticles possessing high saturation magnetizations (50–80 emu/g) and high r_2_ values [67,68].

## 5. In Vivo Imaging

### 5.1. Tumor-Targeting T_1_ MRI Contrast Agents

Gd_2_O_3_ nanoparticles are the most widely applied Ln_2_O_3_ nanoparticles as in vivo tumor-targeting T_1_ MRI contrast agents because of the high spin magnetic moment (s = 7/2) of Gd^3+^ (^8^S_7/2_), which is the largest value among the elements in the periodic table and thus provides very high T_1_ MR contrast. Therefore, various studies on the synthesis and application of tumor-targeting Gd_2_O_3_ nanoparticles have been reported, which are discussed below according to the type of tumor-targeting ligands used.

#### 5.1.1. Chlorotoxin (CTX)

**Figure 10:**
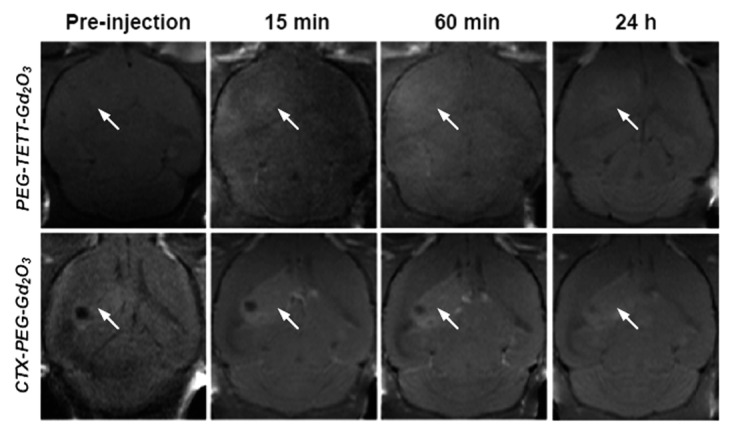
T_1_ MR images of mice brains with C6 glioma before and after (15 min, 60 min, and 24 h) intravenous injection of PEG-TETT-Gd_2_O_3_ and CTX-PEG-TETT-Gd_2_O_3_ nanoparticle samples into mice tails at 7.0 T. Tumor sites are indicated with arrows. Adapted from [45]. Copyright 2014 The Royal Society of Chemistry.

Gu et al. developed a mouse brain tumor-targeting contrast agent using CTX as a tumor-targeting ligand [45]. CTX is a peptide consisting of a 36-amino acid sequence found in the venom of the deathstalker scorpion [69]. CTX preferentially binds to glioma cells, enabling the development of therapeutic and diagnostic protocols for several types of tumors [70]. In this study, CTX-PEG-TETT-coated Gd_2_O_3_ nanoparticles with a core diameter of 3.46 nm and r_1_ value of 8.41 s^−1^ mM^−1^ at 7.0 T were applied to tumor-targeting imaging in vivo. Figure 10 shows a series of in vivo T_1_ MR images of mice with brain glioma tumor after mice tail intravenous injection of PEG-TETT-Gd_2_O_3_ and CTX-PEG-TETT-Gd_2_O_3_ nanoparticle samples at the same Gd dose (6 mg Gd/kg). The PEG-TETT-Gd_2_O_3_ nanoparticles slightly improved the contrast of glioma sites (labeled with arrows) compared with the preinjection image. However, the CTX-PEG-TETT-Gd_2_O_3_ nanoparticles exhibited highly enhanced T_1_ MR images in brain tumors (labeled with arrows) after injection, clearly supporting that the CTX-PEG-TETT-Gd_2_O_3_ nanoparticles act as a tumor-targeting T_1_ MRI contrast agent.

#### 5.1.2. Cyclic RGD (cRGD)

**Figure 11:**
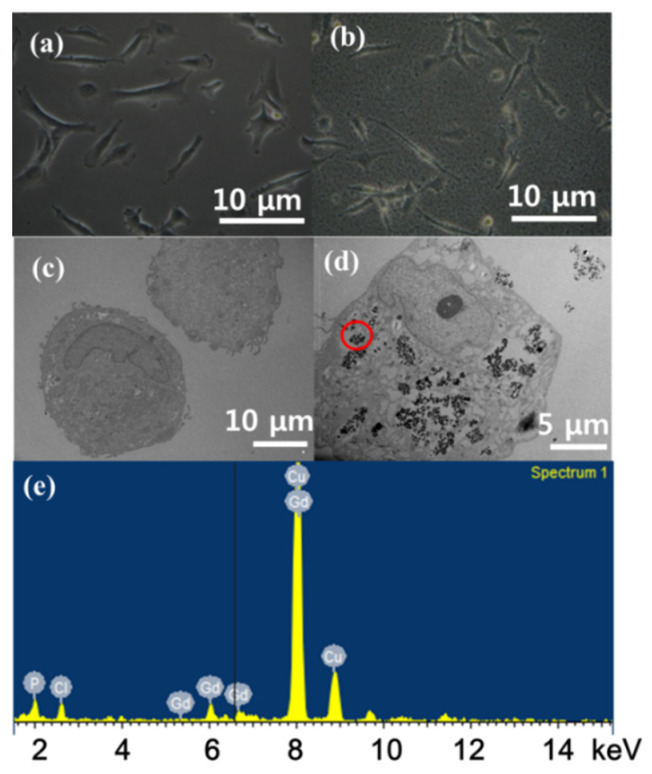
Optical microscope images of U87MG tumor cells incubated (**a**) without and (**b**) with cRGD-Gd_2_O_3_ nanoparticles. HRTEM images of U87MG tumor cells incubated (**c**) without and (**d**) with cRGD-Gd_2_O_3_ nanoparticles. (**e**) EDX spectrum of the circled region in (**d**). Adapted from [47]. Copyright 2018 Wiley.

Ahmad et al. investigated Gd_2_O_3_ nanoparticles that were directly conjugated with cRGDs [47]. Prior to measuring in vivo T_1_ MR images to confirm tumor targeting, an in vitro cellular incubation experiment was conducted with cRGD-Gd_2_O_3_ nanoparticles to confirm the internalization of the nanoparticles in the tumor cells, which is particularly important in the case of tumor theragnosis. To investigate this, human brain glioma (U87MG) tumor cells were incubated with cRGD-Gd_2_O_3_ nanoparticles (0.01 mM Gd). Untreated cells as a control group are shown in Figure 11a. The treated cells exhibited different surface morphologies from that of the control cells due to nanoparticle attachment on the tumor cell surfaces (Figure 11b). The internalization of cRGD-Gd_2_O_3_ nanoparticles in the tumor cells was confirmed by analyzing the TEM images of the cells, where no cRGD-Gd_2_O_3_ nanoparticles were found in the TEM image of the control cells (Figure 11c), whereas they were found in the treated tumor cells (Figure 11d). In addition, an energy-dispersive X-ray (EDX) spectroscopy analysis of the circled region in Figure 11d confirmed the presence of Gd (Figure 11e), supporting the internalization of cRGD-Gd_2_O_3_ nanoparticles in the U87MG tumor cells.

**Figure 12:**
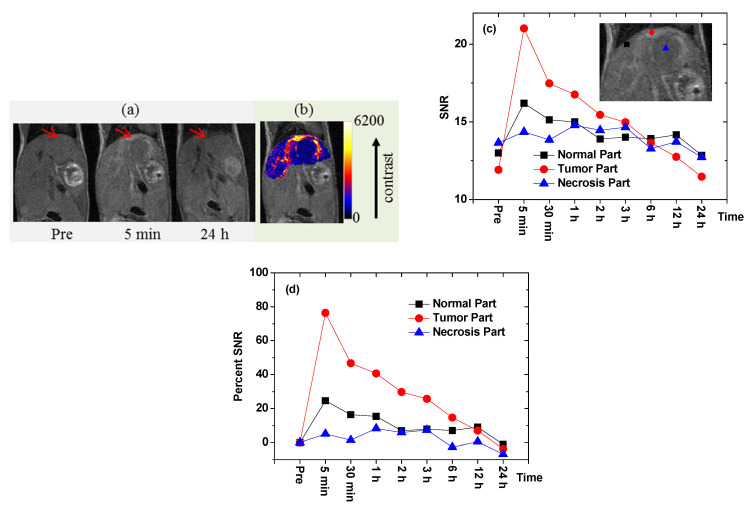
(**a**) T_1_ MR images of a U87MG tumor-bearing nude mouse in the liver before and after intravenous injection (arrows indicate the tumor region). (**b**) Color map of the middle T_1_ MR image in (**a**), showing the widely spread liver tumor (brighter region). (**c**) Plots of the SNRs of the ROIs in the normal, tumor, and necrosis parts of the liver versus time. (**d**) Plots of the percentage SNRs in the normal, tumor, and necrosis parts of the liver versus time; percentage SNR = {[SNR (time)-SNR (Pre)]/SNR (Pre)} × 100. Adapted from [47]. Copyright 2018 Wiley.

To demonstrate the occurrence of in vivo tumor targeting via T_1_ MRI, a sample solution containing cRGD-Gd_2_O_3_ nanoparticles was injected into the tail vein of a U87MG tumor-bearing mouse inoculated into the liver (injection dose = ~0.1 mmol Gd/kg) [47]. T_1_ MR images were obtained before and after injection (Figure 12a). As shown in the figure, tumor targeting was confirmed via positive contrast enhancement in the liver tumor 5 min after injection, indicating the accumulation of the nanoparticles in the tumor. Figure 12b shows a color map of the middle T_1_ MR image depicted in Figure 12a, in which the contrast enhancement in the liver tumor (i.e., the widely spread brighter part) can be clearly observed. The signal-to-noise ratios (SNRs) of the three regions of interest (ROIs) in the liver, i.e., the normal region (filled square), the tumor region (filled circle), and the necrotic region (filled triangle), as labeled in the inserted T_1_ MR image, are plotted versus time in Figure 12c. The T_1_ contrast enhancement in the liver tumor was approximately three times higher than in the normal region of the liver, as shown in the percentage SNR plots in Figure 12d. These results suggest that the cRGD-Gd_2_O_3_ nanoparticles act as a tumor-targeting T_1_ MRI contrast agent.

#### 5.1.3. TAT Peptide

Ahmad et al. explored the tumor-imaging application of transactivator of transcription (TAT) peptide-conjugated Gd_2_O_3_ nanoparticles [48]. The TAT peptide, which contains 48−57 fragments of the basic domain of the human immunodeficiency virus type 1 TAT protein, exhibits cell-penetrating properties [71]. To date, various nanomaterials grafted with TAT peptides have been applied in gene delivery, drug delivery, and tumor-cell imaging [72,73,74]. Ahmad et al. observed a greater accumulation of TAT peptide-grafted Gd_2_O_3_ nanoparticles in tumor cells than in normal cells in a mouse via T_1_ MRI in vivo [48]. The TAT peptide allowed cell penetration of the nanoparticles during circulation through angiogenesis via the EPR effect [26,75].

#### 5.1.4. Linear RGD

**Figure 13:**
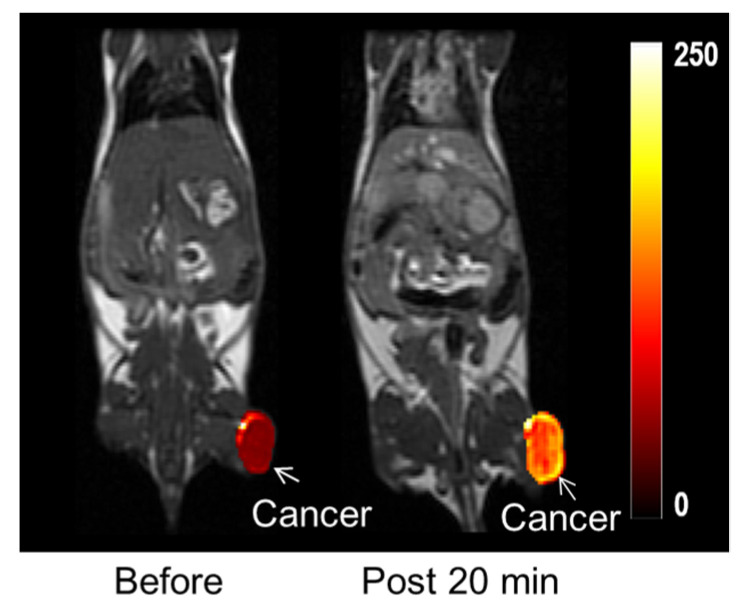
T_1_ MR images before and 20 min after (labeled “post 20 min”) intravenous injection into the mouse tail: the bright contrast at the tumor (labeled with arrows) was due to the accumulation of nanoparticles at the tumor site. Adapted from [44]. Copyright 2020 The Royal Society of Chemistry.

Recently, Ho et al. synthesized highly stable Gd_2_O_3_ nanoparticles coated with PAA and then conjugated with liner RGD (Figure 2) to target tumors by binding to α_v_β_3_ and α_v_β_5_ integrins, which are overexpressed in tumor angiogenic sites and tumor cells [44]. The RGD-PAA-Gd_2_O_3_ nanoparticles accumulated at the tumor sites, showing enhanced T_1_ contrast at the tumor sites after injection (Figure 13). This result was probably due to the combination of various outstanding properties of the RGD-PAA-Gd_2_O_3_ nanoparticles, such as the delivery of hundreds of Gd atoms per nanoparticle to the tumor site, tumor-targeting ability, and ultrasmall size, which provides them with good transport properties during circulation through blood vessels and tumor-cell penetration. This study indicated that the RGD-PAA-Gd_2_O_3_ nanoparticles are applicable to in vivo tumor diagnosis.

### 5.2. MRI-Guided Therapy

**Figure 14:**
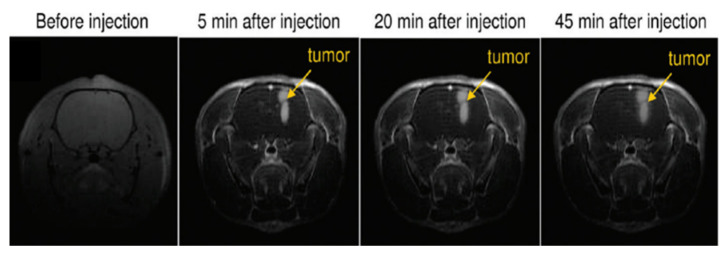
T_1_-weighted images of the brain of a rat having intracerebral 9L gliosarcoma (9LGS) before and after (5, 20, and 45 min) intravenous injection of gadolinium-based nanoparticles. Adapted from [76]. Copyright 2011 American Chemical Society.

One of the main advantages of Gd_2_O_3_ nanoparticles as T_1_ MRI contrast agents is that they can be used for MRI-guided therapy. For example, Le Duc et al. developed gadolinium-based nanoparticles encapsulated in a polysiloxane shell and then grafted with diethylenetriaminepentaacetic dianhydride for nanoparticle colloidal stabilization [76]. They used the nanoparticles for tumor therapy using X-ray microbeam radiation [77,78], which improved the survival of mice against an aggressive brain tumor. The efficacy of this treatment depended on how accurately MRI contrast agents locate the tumor position and shape. In the T_1_ MR images recorded a few minutes after intravenous injection, the delineation of the tumor was clearly distinguished from normal tissues in a rat brain as shown in Figure 14, and X-ray microbeam radiation was then conducted to kill the brain tumor cells.

### 5.3. T_2_-Weighted MRI

**Figure 15:**
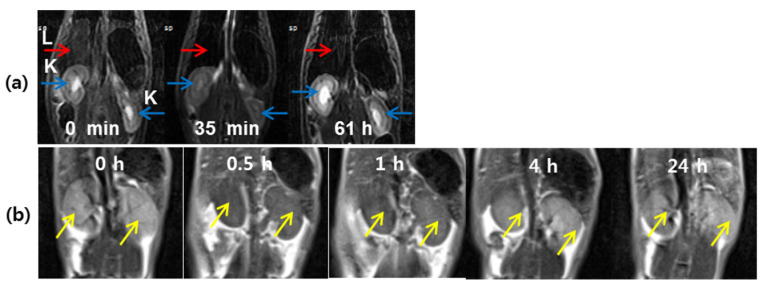
(**a**) A series of 3.0 T in vivo T_2_ MR images: coronal views of kidneys and liver (labeled as “K” and “L”, respectively) before and after intravenous injection of an aqueous solution of D-glucuronic acid-coated Dy_2_O_3_ nanoparticles into the mouse tail [79]. (**b**) In vivo coronal 3.0 T T_2_ MR images of the mice kidneys (indicated with arrows) as a function of time before and after intravenous injection of an aqueous solution of carbon-coated Dy_2_O_3_ nanoparticles into the mouse tail [80]. Adapted from [79,80]. Copyrights 2012 Elsevier and 2020 MDPI.

Recently, Ln_2_O_3_ (Ln = Tb, Dy, and Ho) nanoparticles with appreciable r_2_ and negligible r_1_ values, suitable for T_2_ MRI were developed [42,43,50,66,79,80]. For example, PAA-coated Ln_2_O_3_ (Ln = Tb and Ho) nanoparticles exhibited appreciable r_2_ values at 3.0 T MR field (3.19 s^−1^ mM^−1^ for Ln = Tb and 1.44 s^−1^ mM^−1^ for Ln = Ho), enhanced r_2_ values at 9.4 T MR field (16.40 s^−1^ mM^−1^ for Ln = Tb and 9.20 s^−1^ mM^−1^ for Ln = Ho) [43], and negligible r_1_ values at all MR fields. With such r_1_ and r_2_ values, only the exclusive induction of T_2_ relaxations by the nanoparticles occurs at all MR fields. The appreciable T_2_ MR contrast enhancements at 9.4 T MR field confirmed the effectiveness of the nanoparticles as T_2_ MRI contrast agents at high MR fields in vivo [43]. Dy_2_O_3_ nanoparticles constitute another example with application in T_2_ MRI. Thus, D-glucuronic acid-coated Dy_2_O_3_ nanoparticles exhibited T_2_ MR contrast enhancements in a mouse liver at 3.0 T MR field in vivo [79] (Figure 15a), proving the potential of Dy_2_O_3_ nanoparticles as T_2_ MRI contrast agents. Recently, Yue et al. investigated carbon-coated Dy_2_O_3_ nanoparticles as a new class of T_2_ MRI contrast agent at 3.0 T MR field [80]. The nanoparticles were nearly non-toxic via an in vitro cellular cytotoxicity assay. The presence of numerous hydroxyl groups on the carbon-coating layer conferred the colloidal nanoparticles with stability in aqueous media. The nanoparticles exhibited T_2_ contrast enhancement in the mice kidneys after intravenous administration, acting as a T_2_ MRI contrast agent (Figure 15b).

### 5.4. CT Imaging

**Figure 16:**
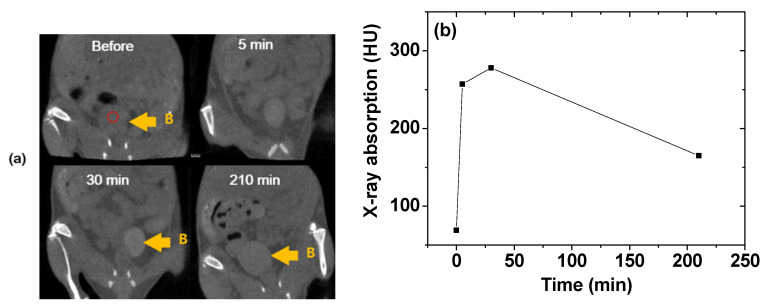
(**a**) In vivo CT images of a mouse bladder (labeled B) and (**b**) plot of the X-ray absorption of the ROI in the bladder (indicated by the small, dotted circle in (**a**)) before and after intravenous injection of an aqueous sample into the mouse tail [81]. Adapted from [81]. Copyright 2015 Springer Nature.

Lanthanide elements exhibit higher X-ray attenuation coefficients [16] than iodine, which is currently used as a CT contrast agent in its organic molecular forms. Consequently, Ln_2_O_3_ nanoparticles can be applied as CT contrast agents [17,18,81,82]. For example, an aqueous solution of Gd_2_O_3_ nanoparticles coated with iodine compounds was used to investigate CT imaging in vivo [81]. Brighter contrast enhancements were observed in mouse bladder (labeled B in Figure 16a) after intravenous tail injection. The X-ray absorption in the ROI of the bladder (indicated by a small, dotted circle in Figure 16a) indicated that the contrast reached a maximum at ~30 min after injection and then decreased with time (Figure 16b), suggesting that the sample solution was excreted through the bladder as urine. This result showed that an aqueous solution of Gd_2_O_3_ nanoparticles might serve as a CT contrast agent.

## 6. Ln_2_O_3_ Nanoparticle Toxicity

**Figure 17:**
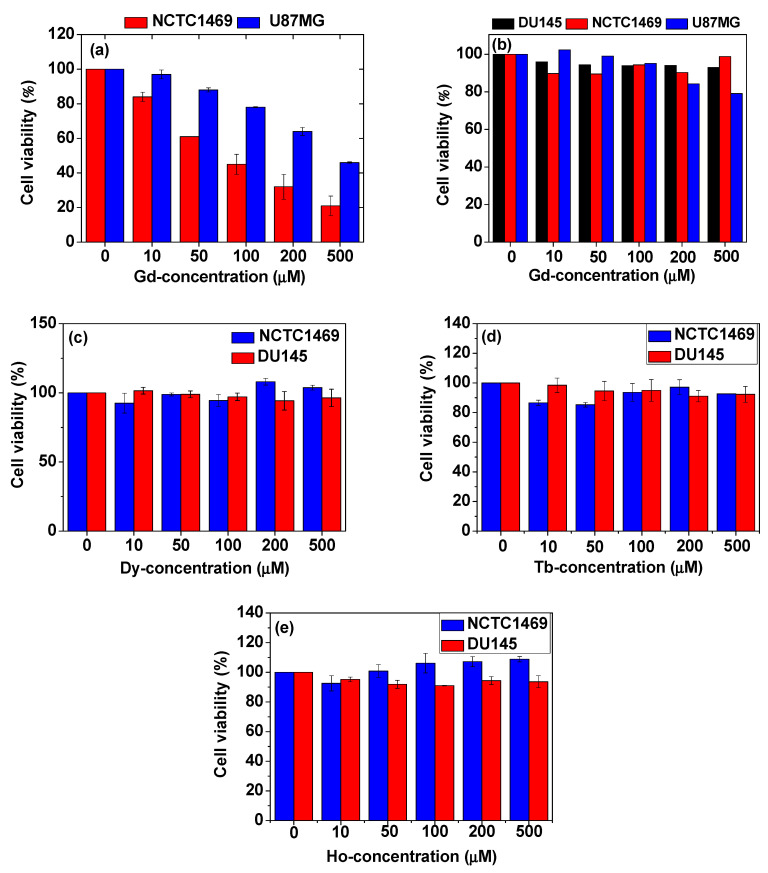
In vitro cytotoxicities of (**a**) uncoated Gd_2_O_3_ nanoparticles in NCTC1469 and U87MG cell lines and (**b**) PAA-coated Gd_2_O_3_ nanoparticles in DU145, NCTC1469, and U87MG cell lines [83]. PAA-coated Ln_2_O_3_ nanoparticles (Ln = (**c**) Dy, (**d**) Tb, and (**e**) Ho) in DU145 and NCTC1469 cell lines [42,43]. Adapted from [42,43,83]. Copyrights 2018, 2020 and 2021 The Royal Society of Chemistry, Wiley & MDPI.

As shown in Figure 17a, bare Gd_2_O_3_ nanoparticles exhibit toxicities in both NCTC1469 normal and U87MG tumor-cell lines, whereas PAA-coated Gd_2_O_3_ nanoparticles are nearly non-toxic up to 500 μM Gd with cell viabilities of ∼93% in DU145, ∼99% in NCTC1469, and ∼80% in U87MG cell lines (Figure 17b) [83]. Other PAA-coated Ln_2_O_3_ nanoparticles (Ln = Dy, Tb, and Ho) also exhibit very low cytotoxicities in both DU145 and NCTC1469 cell lines (Figure 17c–e) [42,43], showing good biocompatibilities. These examples demonstrate that Ln_2_O_3_ nanoparticles must be well protected with water-soluble and biocompatible ligands for biomedical applications.

Lanthanides are relatively non-toxic elements [37,38]. For example, for lanthanide chlorides, the lethal dose causing the death of 50% of a group of 10 animals (LD50) is higher than 10 and 450 mg per kg bodyweight for intravenous and intraperitoneal injections, respectively [84]. In the case of Ln_2_O_3_ nanoparticles, other properties, either alone or in concert, must be considered to evaluate the possible toxic effects of the nanoparticles. These properties include chemical composition, doping, hydrodynamic size, shape, redox properties, tendency for aggregation, composition of the shell or coating material, surface modifications, colloidal stability, solubility, biodegradability, concentration, and duration of exposure [85]. Although several studies have investigated the toxicity of lanthanide-based nanoparticles having different properties, such as chemical composition, size, surface ligands, and lanthanide concentration [86,87,88], the lack of literature data has prevented drawing firm conclusions for the assessment of the potential toxicity of Ln_2_O_3_ nanoparticles. Furthermore, several aspects of the biological interaction of Ln_2_O_3_ nanoparticles in living systems still need to be unveiled. For example, despite in vitro cytotoxicity studies being conducted using various cell cultures, these studies did not provide information about long-term safety and cytotoxicity [89,90], for which in vivo cytotoxicity studies would be needed.

Nevertheless, several important conclusions can be extracted from the currently available literature data. In the case of biomedical applications, Ln_2_O_3_ nanoparticles are usually introduced into the body by intravenous injections and circulated by the bloodstream primarily to organs, such as the liver and spleen, kidneys, heart, lungs, and brain [91]. The possible retention or uptake in blood and organs strongly depends on the surface properties of the nanoparticles. Ligand coating can promote the interaction of the Ln_2_O_3_ nanoparticles with the cell membranes, favoring the internalization of the nanoparticles by various types of cells. However, biologically inert coating ligands, such as PEG, may prolong the circulation in the bloodstream of nanoparticles [11]. In addition, nanoparticle size is an important factor for the excretion route [92,93]. Thus, nanoparticles smaller than 3 nm can be excreted by renal filtration [19,20], whereas those larger than 3 nm are enclosed by a phagocyte system. As a part of the immune system, the phagocyte system is composed of several types of phagocytic cells in the reticular tissue within the body, whose main function is to remove undesired species, such as bacteria, viruses, and foreign materials, including nanoparticles.

**Figure 18:**
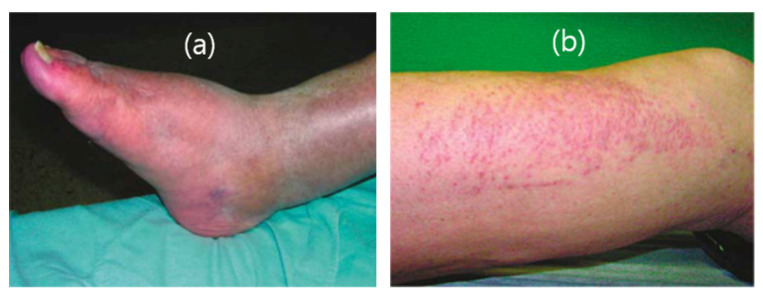
Clinical pictures of the legs of two patients with NSF for (**a**) about three years and (**b**) four weeks. Adapted from [99]. Copyright 2007 International Society of Nephrology.

The toxicity of Ln_2_O_3_ nanoparticles differs depending on the lanthanide ions. For example, Er_2_O_3_ shows higher toxicity than Gd_2_O_3_ and La_2_O_3_ and is highly toxic to zebrafish embryos at a concentration of 50 ppm Er, causing significant mortality and morphological malformations [93]. Meanwhile, the toxicity of Gd_2_O_3_ nanoparticles is primarily attributed to the release of Gd^3+^ ions [94,95,96]. The toxicity of Gd^3+^ ions has been addressed not only for Gd_2_O_3_ nanoparticles but also for a variety of molecular Gd^3+^-chelates. In the case of Gd_2_O_3_ nanomaterials, Eu^3+^-doped Gd_2_O_3_ (Gd_2_O_3_:Eu^3+^) nanotubes can adversely affect bone marrow stromal cells (BMSCs) [97]. Yang et al. reported the application of ^153^Sm-doped Gd(OH)_3_ nanorods as a potential MRI contrast agent [98]. In vitro cell toxicity tests revealed that Gd(OH)_3_ nanorods have no toxic effect on cellular proliferation and viability. Furthermore, an in vivo toxicity test using Kunming mice showed that injection up to 100 mg/kg of Gd(OH)_3_ nanorods had no toxic effect up to 150 days after exposure. However, this in vivo test investigated the short-term toxic effect of Gd(OH)_3_ nanorods. In contrast, the long-term toxicity of clinically used Gd^3+^-chelates was reported. When used in patients with severely compromised kidney function, Gd^3+^-chelates promoted the development of nephrogenic systemic fibrosis (NSF), which is a rare disease affecting different parts of the body that can lead to thickening or hardening of the skin and deposits [99] (Figure 18). Moreover, NSF is a progressive condition that can be fatal because it may cause multiple organ failure [99,100,101,102]. Gd^3+^ retention in the body, which is greater with linear structured Gd^3+^-chelates than with macrocyclic structured Gd^3+^-chelates, was demonstrated to be associated with NSF.

Compared with clinically used Gd^3+^-chelates, the toxicity of Ln_2_O_3_ nanoparticles, including Gd_2_O_3_ nanoparticles, has been less explored. For instance, the extent, mechanism, chemical form, and clinical implications of chronic lanthanide retention for Ln_2_O_3_ nanoparticles remain unknown. Therefore, more comprehensive investigations are required to improve our understanding of Ln_2_O_3_ nanoparticle toxicity and its clinical importance. In this context, the development of new experimental techniques may play a significant role in unveiling lanthanide nanotoxicity. For example, nanotoxicogenomics [103], which uses DNA microarray technologies to investigate the impact of nanoparticles on global gene expression profiles of cells and tissues, has emerged as a new field of toxicology to provide new and important insights into the toxicity of Ln_2_O_3_ nanoparticles.

## 7. Conclusions and Perspectives

Recent studies on the synthesis, surface modification, tumor-targeting ligand conjugation, toxicity, and novel biomedical applications to tumor-targeting T_1_ MRI and image-guided tumor therapy of Ln_2_O_3_ nanoparticles were reviewed here. In addition, T_2_ MRI and CT imaging applications were also discussed. High-quality Ln_2_O_3_ nanoparticles can be synthesized via the polyol method and organic-phase thermal decomposition method and then surface-coated with hydrophilic and biocompatible ligands for colloidal stability and biocompatibility. Polymers are more efficient than small molecules as surface-coating ligands because of their many –COOH, –NH_2_, –OH groups, which can be attached to nanoparticles and conjugated with tumor-targeting ligands and drugs.

The interest in Ln_2_O_3_ nanoparticles in the field of medical imaging lies principally in the excellent imaging properties of lanthanides, which arises from their appreciable paramagnetic moments at room temperature; Gd^3+^ has the highest 4f-electron spin magnetic moment (s = 7/2) among the elements in the periodic table, which is extremely useful for T_1_ MRI, and other Ln^3+^ ions (Ln = Tb, Dy, and Ho) have a very high 4f-electron spin–orbital magnetic moment, which is suitable for T_2_ MRI. In addition, lanthanide elements possess higher X-ray attenuation coefficients than iodine, which is currently used as a CT contrast agent in its organic compound forms. The potential of Ln_2_O_3_ nanoparticles as CT contrast agents has been confirmed by recording in vivo CT images. In the case of tumor targeting, Gd_2_O_3_ nanoparticles are the most widely investigated Ln_2_O_3_ nanoparticles due to their very high T_1_ MRI sensitivity. Various tumor-targeting ligands have been conjugated to Gd_2_O_3_ nanoparticles for tumor imaging and T_1_ MRI-guided tumor therapy. Especially, the enhanced accumulation of tumor-targeting ligand-coated Gd_2_O_3_ nanoparticles at tumor cells compared with that at normal cells allowed the development of precise image-guided tumor therapies via clear distinction and delineation of tumors from normal tissues.

As reviewed here, further intensive research is essential to achieve the ultimate goal of using Ln_2_O_3_ nanoparticles for tumor-targeting diagnosis and therapy and nontumor-targeting medical imaging. In addition, sophisticated toxicological and pharmacological improvements are required to demonstrate the safety of nanoparticle formulations prior to clinical trials. We hope that this review will guide the future development of biomedical applications of Ln_2_O_3_ nanoparticles.

## Figures and Tables

**Table 1:** Summary of physicochemical characterization.

Properties	Technique ^1^	Properties	Technique ^1^
Particle diameter	HRTEM	Hydrodynamic diameter	DLS
Crystal structure	XRD	Ligand surface coating	FT-IR absorption, EA
Ligand surface-coating amount	TGA, EA	Metal concentration in water	ICPAES
Cellular cytotoxicity	MTT, WST	Magnetization	VSM
Relaxivity (r_1_ and r_2_)	MRI	MR image	MRI
X-ray attenuation power	CT	CT image	CT

^1^ HRTEM = high-resolution transmission electron microscope; DLS = dynamic light scattering; XRD = X-ray diffraction; FT-IR = Fourier transform-infrared; EA = elemental analysis; TGA = thermogravimetric analysis; ICPAES = inductively coupled plasma atomic emission spectrometry; MTT = methylthiazoly tetrazolium; WST = water-soluble tetrazolium salt; VSM = vibrating sample magnetometer; MRI = magnetic resonance imaging; CT = computed tomography.

## Data Availability

Not applicable.
